# Immunoinformatics study to explore dengue (DENV-1) proteome to design multi-epitope vaccine construct by using CD4+ epitopes

**DOI:** 10.1186/s43141-023-00592-9

**Published:** 2023-11-21

**Authors:** Nishat Bano, Ajay Kumar

**Affiliations:** Department of Biotechnology, Faculty of Engineering and Technology Rama University, G.T. Road, Kanpur, 209217 India

**Keywords:** Dengue virus, Immunoinformatics, Vaccine construct, CD4+ epitopes, MD simulation

## Abstract

**Background:**

Immunoinformatics is an emerging interdisciplinary field which integrates immunology, bioinformatics, and computational biology to study the immune system. In this study, we apply immunoinformatics approaches to explore the dengue proteome in order to design a multi-epitope vaccine construct.

**Methods:**

We used existing databases and algorithms to predict potential epitopes on dengue proteins and used a bioinformatics approach to identify the most promising epitopes. We then used molecular modelling to develop a multi-epitope construct which could be used as a potential vaccine. The results of this study demonstrate that immunoinformatics is a powerful tool for exploring and designing potential vaccines for infectious diseases like dengue.

**Results:**

Here, we found four CD4+ epitopes NLKYSVIVTVHTGDQ, ANPIVTDKEKPVNIE, LDPVVYDAKFEKQL, and VGAIALDFKPGTSGS that were used to design vaccine construct. The vaccine construct docked with TLR5. RMSD values suggest that docked complex of TLR5 and vaccine construct have putative stable interaction to induce immunogenic effects on host.

**Conclusions:**

Furthermore, our study provides a proof of concept for the use of immunoinformatics approaches in DENV vaccine design. This vaccine can be effective in treating patients infected with DENV virus.

## Background

Immunoinformatics is an emerging field that combines bioinformatics, immunology, and computational biology to develop tools and technologies to study the immune system [1–4]. Recently, it has been used to study the proteome of dengue virus, in order to design a multi-epitope vaccine construct. This introduction will provide an overview of immunoinformatics and its use to explore the dengue proteome, in order to design a multi epitope vaccine construct [5–7]. Furthermore, the advantages and challenges of immunoinformatics in designing such a construct will also be discussed. Dengue virus is a mosquito-borne virus that causes severe debilitating illnesses in humans, such as dengue fever, dengue hemorrhagic fever, and dengue shock syndrome. Dengue is a mosquito-borne viral infection that has become a major global health concern in recent years. The virus is spread through the bite of an infected *Aedes* species mosquito, which are found in tropical and subtropical climates. The disease can cause a wide range of symptoms, from mild fever and body aches to more severe complications such as hemorrhagic fever and even death [8–10]. The World Health Organization (WHO) estimates that there are over 390 million dengue infections each year, with the greatest burden of disease impacting people in the Asia-Pacific region. As the *Aedes* mosquitoes that spread the virus are found in many parts of the world, the virus has the potential to spread to other regions with suitable conditions. Dengue prevention relies heavily on controlling the mosquito population, which is best done through a combination of practices. These include using insect repellent, wearing protective clothing, regularly changing standing water, and using insecticides. It is also important to encourage people in at-risk areas to get tested and treated if necessary, as early diagnosis and treatment can help to reduce the severity of the disease. In addition to prevention efforts, research is being conducted to develop a vaccine for dengue. Although no vaccine is currently available, the hope is that one could be developed in the near future to help reduce the spread of the disease [11–13]. The dengue virus is a single-stranded, positive-sense, enveloped RNA virus. It is composed of a protein shell (capsid) made of three proteins (C, prM, and E) that encloses the viral genome. The capsid is surrounded by a lipid envelope that is decorated with two types of glycoprotein spikes (M and E). The M and E glycoproteins serve as receptors for the virus to attach to and enter host cells. The virus particles measure approximately 50–60 nm in diameter.

Dengue virus classification is based on the characteristics of the four distinct serotypes (DENV-1, DENV-2, DENV-3, and DENV-4). Each serotype is classified as a member of the Flaviviridae family, which also includes other medically important viruses such as yellow fever, West Nile, and Zika. The dengue is a serious public health concern that requires a comprehensive approach to control and prevent. This includes controlling mosquito populations through appropriate control methods, encouraging people to get tested and treated if necessary, and researching a vaccine to help reduce the spread of this disease [14, 15]. Therefore, it is essential to develop an effective vaccine to protect people from the virus. Using immunoinformatics, researchers can explore the dengue virus proteome in order to design a multi-epitope vaccine construct. This construct would contain multiple short peptides, or epitopes, derived from the virus proteome, which could stimulate the body’s immune response against the virus. The immunoinformatics process involves analyzing the virus proteome to identify specific epitopes that are then incorporated into a multi-epitope construct. The first step in designing a multi-epitope construct is to identify the relevant epitopes from the virus proteome. This can be done using bioinformatics tools such as sequence and structure analysis, antigen-antibody modelling, and motif identification [16–20]. Once the relevant epitopes have been identified, they can be incorporated into a multi-epitope construct and tested for its ability to stimulate the body’s immune response. The immunoinformatics approach to designing a multi-epitope vaccine construct for dengue virus is a promising and cost-effective way to protect people from the virus. It is a relatively new approach, but it has the potential to revolutionize the way vaccines are designed and has the potential to save thousands of lives every year.

## Methods

### Protein selection for DENV-1

 In silico protein selection from DENV-1 can be accomplished by using a variety of computational methods. These methods include sequence alignment, protein structure prediction, protein-protein interaction analysis, and molecular dynamics simulations. In sequence alignment, the amino acid sequences of proteins from different DENV-1 strains can be compared to identify potential candidates for further study (Table 1).

**Table 1:** Considered proteins for selection of epitopes

NCBI-accession no.	Protein name	No. of amino acids
QQC97218.1	Polyprotein (dengue virus type 1)	3392
AFN87865.1	Envelope protein, partial (dengue virus type 1)	497

### Epitopes prediction from DENV-1

Epitope prediction from DENV-1 can be accomplished using a variety of computational methods. One such method is the computational analysis of the DENV-1 amino acid sequence to identify potential antigenic sites. This can be done using bioinformatics tools such as BLAST or ClustalW. These tools allow researchers to compare the DENV-1 sequence to known protein structures and identify regions of similarity that could potentially be antigenic sites. Tools such as BepiPred can also be used to predict the likelihood of a given region of the DENV-1 sequence being an antigenic site. Alternatively, researchers can also use antigenic mapping tools such as NetMHC to generate detailed predictions of the most likely antigenic sites of DENV-1. Here, we selected T-cell epitopes (CD4+) by using NetMHC server [21, 22]. NetMHC is a bioinformatics server used to predict binding affinities of peptides to the major histocompatibility complex (MHC). It can predict binding affinities of peptides for MHC class I molecules and for MHC class II molecules. It can also predict peptide binding to MHC molecules of other species.

### Multi-epitope vaccine construction and structure prediction

Epitope-based vaccines are typically constructed by combining multiple antigenic peptides that represent a diverse range of epitopes from a pathogen. The composition of the vaccine typically includes peptides from both the conserved and variable regions of a pathogen’s proteome, which allows for the immunogenicity of the vaccine to be maximized. Once the composition of the vaccine has been determined, the structure of the vaccine must be predicted. This is typically done through computer-aided molecular design (CAMD). CAMD algorithms are used to predict the structure of the vaccine by analyzing the sequence of the peptides, predicting the three-dimensional structure of the peptide sequence, and then combining the individual peptide structures into a single structure. The accuracy of the prediction is dependent upon the quality of the input sequence data. Here, we incorporated all identified CD4+ epitopes with suitable linkers and adjuvants. Once the structure has been predicted, the vaccine can then be synthesized and tested for efficacy. I-TASSER tool can be used to predict the three-dimensional structure of a vaccine. This tool can be used to design a multi-epitope vaccine for a particular pathogen by predicting the three-dimensional structure of the antigens present in the pathogen. I-TASSER uses a combination of sequence and structure comparison methods to predict the best three-dimensional structure for a given protein [23, 24]. The predicted structures can then be used to identify the epitopes present in the pathogen’s antigen and used to construct a multi-epitope vaccine.

### Vaccine structure validation

MolProbity is another software program used to validate the structure of a vaccine. It can be used to analyze the geometric and energy-related properties of a molecule and detect any potential errors in the structure. MolProbity also evaluates the overall stereochemistry of the molecule and can detect any potential steric clashes or other structural problems [25, 26]. Additionally, MolProbity can compare the structure of the molecule to known structures and detect any potential errors or discrepancies.

### Molecular docking of multi-epitope vaccine and TLR5

Molecular docking is a computational method used to predict the binding affinity between a drug molecule and a target protein. It is used to predict the binding mode of the drug molecule on a specific protein target and to determine the strength of the interactions between them. To perform the docking of multi-epitope vaccine and TLR5 (Toll-like receptor 5), HDOCK is a suitable program. HDOCK uses a hybrid approach combining the rigid-body docking search with the flexible side chain optimization [27, 28]. It works by first performing a rigid body docking search to identify potential binding modes and then performing a flexible side chain optimization to refine the binding modes and estimate the binding free energies. The docking parameters such as the ligand, receptor, and scoring functions can be customized to ensure the best results. Once the parameters are configured, HDOCK will run multiple searches and return a set of binding modes with the lowest binding free energies. The user can then examine the output to determine whether the multi-epitope vaccine binds to TLR5 and the strength of the binding. The docking results can also be used to design better multi-epitope vaccines and to further optimize the binding affinity.

### MD simulation for multi-epitope vaccine

The simulation of molecular dynamics for a complicated protein-ligand system in a water solvent system was performed using the glide docking approach. The system was created using Desmond/Maestro Schrödinger’s system builder and consisted of a ten orthorhombic box form [29, 30]. The volume of the system was minimized, and the water solvent was modelled using the simple point charge (SPC) water model. To neutralize the docked complex, nine Cl ions were used, and the system received an additional 0.15 M of Na+ and Cl ions. The entire system was allowed to relax before the molecular dynamics simulation was initiated. The simulation was carried out in an isothermal-isobaric (NPT) ensemble at a temperature of 310 K and a pressure of 1.01325 bar. The simulation time for molecular dynamics was set to 100 ns. The OPLS-AA force field was used for the simulation. During the simulation, the molecular dynamics approach predicted how each atom in the protein-ligand system would move over time based on a broad model of the physics driving interatomic interactions. The output of the simulation can be used to predict the stability, dynamics, and structural changes in the protein-ligand complex. The results obtained from the molecular dynamics simulation can provide valuable insights into the mechanisms underlying the binding of ligands to proteins and other molecular systems.

## Results

### CD4+ epitopes prediction

After selecting envelop and polyprotein of the viral surface, these sequences of proteins subjected to CD4+ epitopes prediction, and ultimately, we got 3863 epitopes for both the proteins (485 for AFN87865.1 and 3378 for QQC97218.1). The best scored epitopes that reveal strong binding with respective MHC class I allelic determinants were shown in Table 2. Also, antigenicity was determined by deploying VaxiJen tool, where we put the threshold > 0.7 favorable for viruses.

**Table 2:**
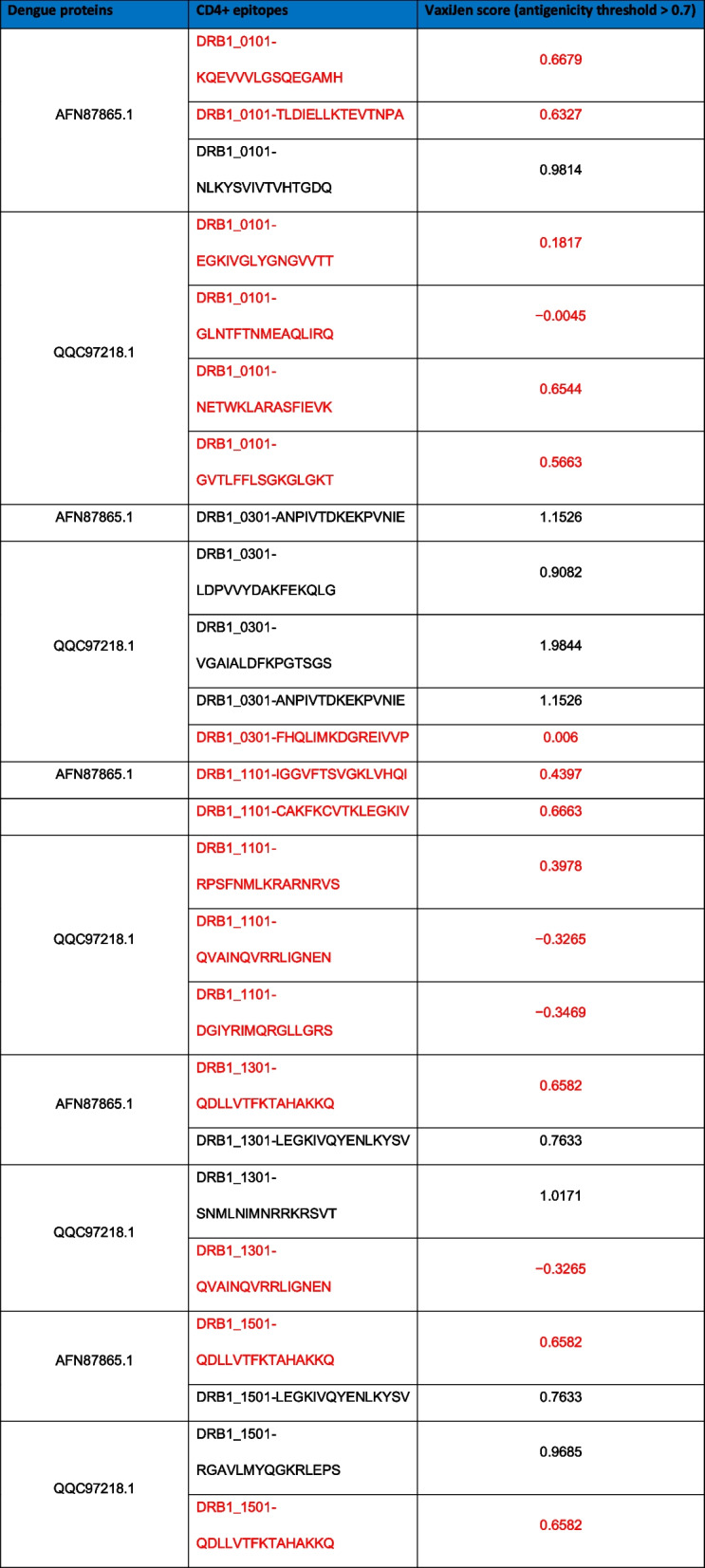
CD4+ best epitopes showing strong binding with MHC allelic determinants, further antigenicity was checked (selected in black/non-selected in red)

### Multi-epitope vaccine construct designing

**Fig. 1:**
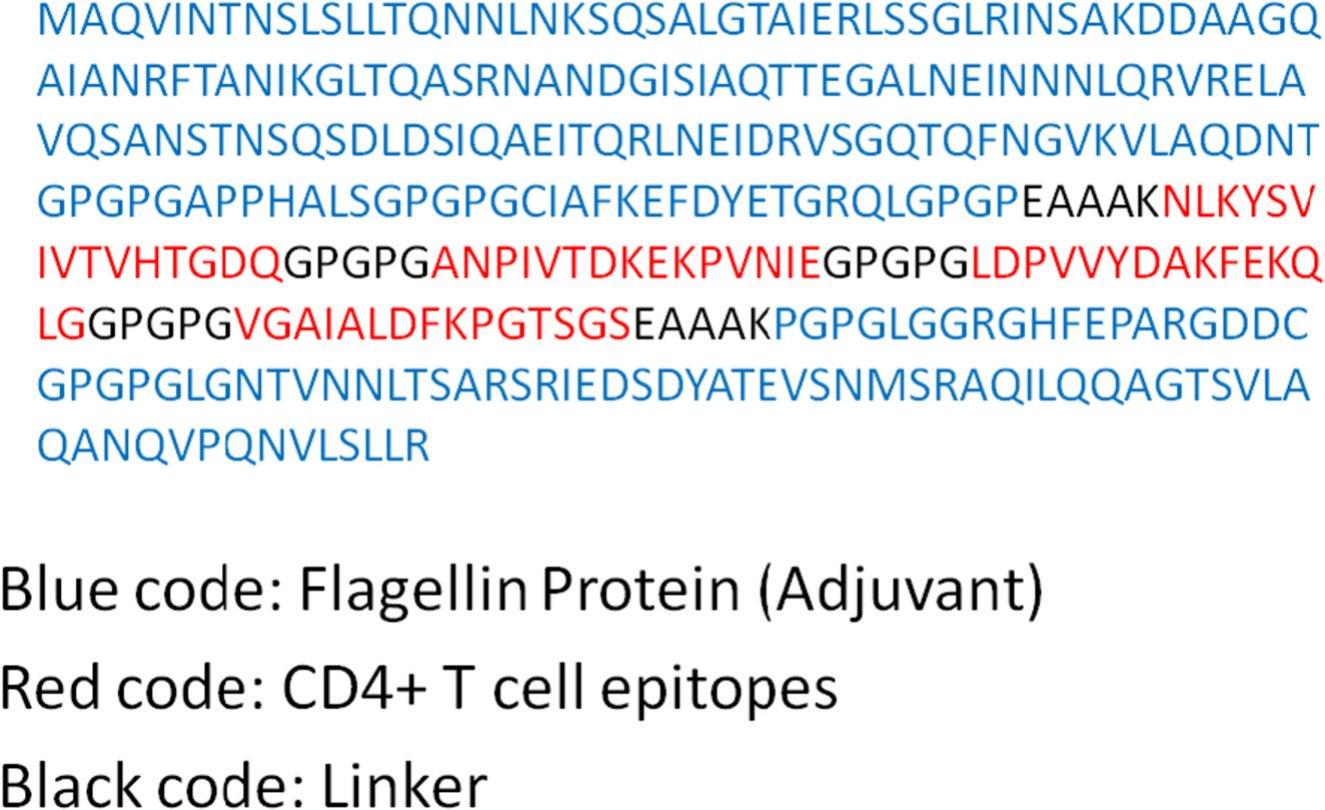
Multi-epitope vaccine construct from CD4+ MHC class II epitopes

Flagellin adjuvant is a novel adjuvant that has been developed to link with CD4+ epitopes. It works by binding to the epitope and forming a stable complex between the epitope and the flagellin. The linker molecules EAAAK and GPGPG are then used to bridge the flagellin and epitope together. This creates a stronger interaction between the flagellin and the epitope, which helps to stimulate the immune system to produce a stronger response to the antigen. The linker molecules also help to stabilize the complex, which increases its effectiveness. This type of adjuvant has been found to be effective in eliciting strong CD4+ responses in animal models and could be a promising adjuvant for future vaccine development (Fig. 1).

### Vaccine structure prediction and validation

**Fig. 2:**
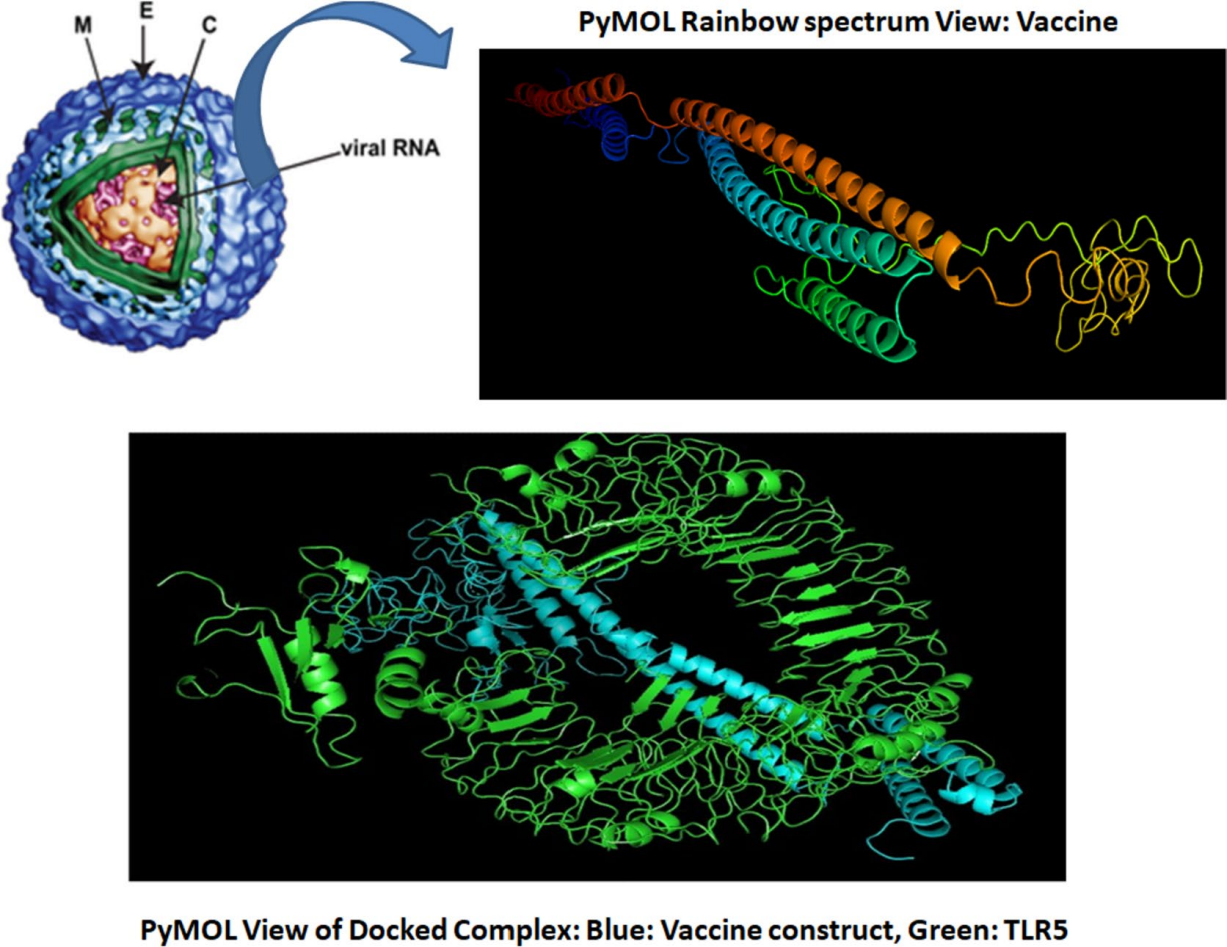
Dengue virus vaccine construct and docked complex PyMOL view

**Fig. 3:**
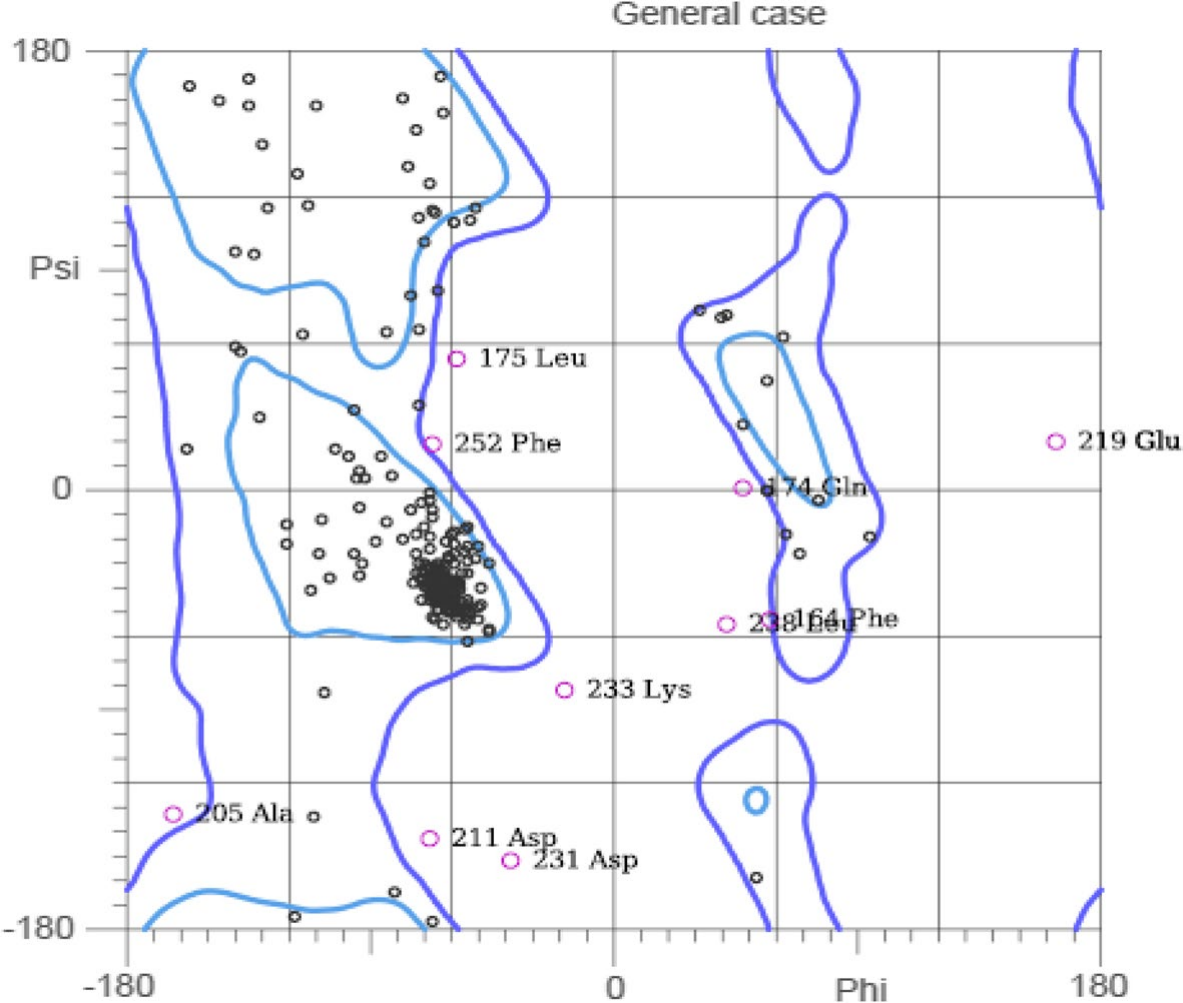
Ramachandran plot for vaccine construct (where 93% residues are in allowed region)

Structure of multi-epitope vaccine was predicted by deploying I-TASSER tool (see Fig. 2). A confidence score, or C-score, is used by I-TASSER to gauge the calibre of projected models. It is determined using the importance of threading template alignments and the convergence parameters from simulations of the structure construction. A model with a high confidence level has a higher C-score and vice versa. The C-score normally falls between −5 and 2. When the native structure is known, the accuracy of structure modelling is often evaluated using the TM-score and RMSD, two well-established benchmarks for comparing the structural similarity of two structures. Here, we estimated C-score = −2.18, estimated TM score = 0.46 ± 0.15, and estimated RMSD = 11.6 ± 4.5Å, for the multi-epitope vaccine, which indicate structural stability. Also, Toll-like receptor 5 (TLR5: PDB ID: 3J0A) was retrieved from RCSB PDB database. Structure of vaccine craft was validated by using MolProbity. The Ramachandran plot is a graphical representation of the conformation of amino acid residues in protein structures. It was named after G. N. Ramachandran, who first proposed the concept in the 1960s. The plot displays the torsional angles of phi (ϕ) and psi (ψ) for each amino acid residue in a protein chain. The ϕ angle is the angle of rotation around the peptide bond, which connects the alpha carbon (Cα) atom of an amino acid residue to the carbonyl carbon atom of the preceding residue. The ψ angle is the angle of rotation around the peptide bond, which connects the Cα atom of an amino acid residue to the nitrogen atom of the succeeding residue. The Ramachandran plot displays the distribution of these two torsional angles for each residue in a protein structure. It is a scatter plot with the ϕ angle on the *x*-axis and the ψ angle on the *y*-axis. The plot is divided into four quadrants that correspond to different conformations: the alpha-helix region, the beta-sheet region, the left-handed alpha-helical region, and the region that corresponds to the non-preferred conformations. The plot is a powerful tool for evaluating the quality of protein structures, as it can reveal potential errors in the protein structure, such as steric clashes, bond lengths, or angles that are outside of the preferred ranges. Here, the plot evaluated from MolProbity server for vaccine construct also provides insight into the stability and folding of protein structures, as it reflects the interactions between the amino acid residues that determine the conformation of the vaccine construct, as out of all residues 93% belongs to allowed regions (see Fig. 3).

### Molecular docking of TLR5 and vaccine construct

Molecular docking was used to predict how a vaccine construct may interact with a target protein, such as a receptor on the surface of immune cells. TLR5 (Toll-like receptor 5) is a receptor protein expressed on the surface of immune cells that recognizes bacterial flagellin, a structural protein found in many bacterial species. Activation of TLR5 by flagellin triggers an immune response that helps to protect the host from bacterial infections. Here, we used DENV-1 epitopes with flagellin in vaccine construct to dock with TLR-5 protein in HDOCK server. The results reveal possible binding of vaccine construct within the TLR-5 binding pocket, with binding energy of −45 Kcal/mol (see Fig. 2).

### Molecular dynamics and simulation

**Fig. 4:**
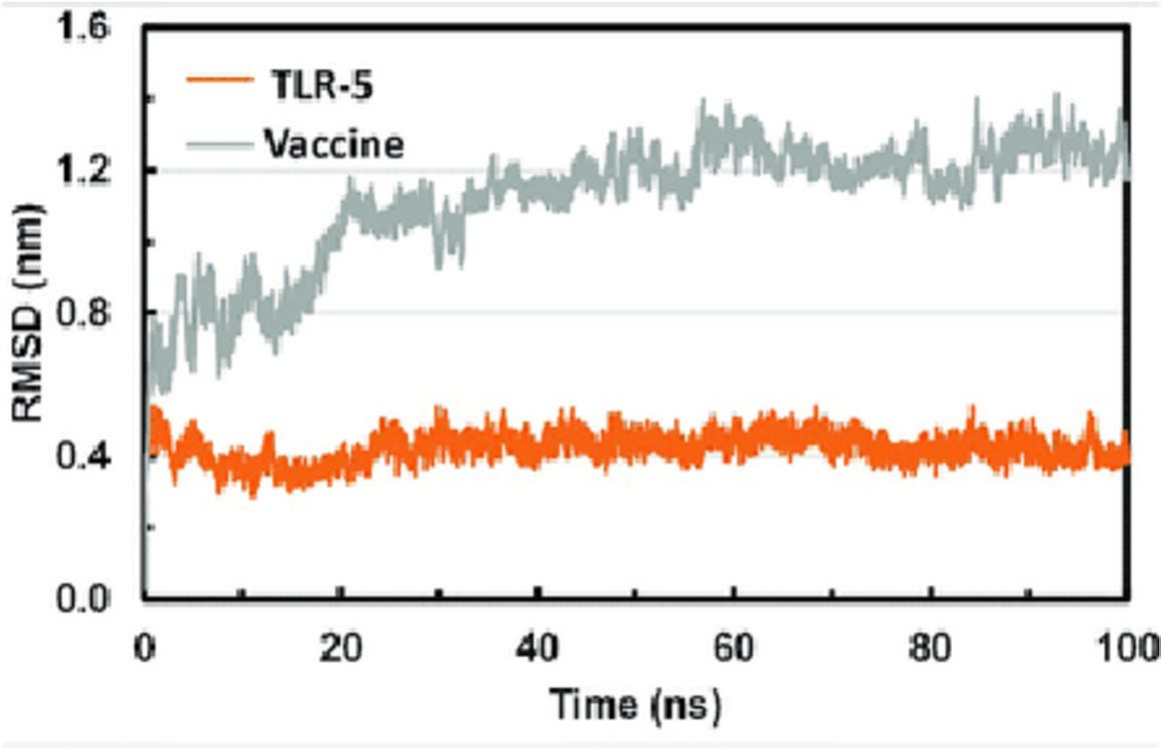
Root-mean-square deviation plot for TLR-5 (orange) and docked vaccine construct (gray)

Based on a broad model of the physics driving interatomic interactions, molecular dynamics (MD) simulations forecast how each atom in a protein or other molecular system will move over time. For the simulation of molecular dynamics, glide docking was employed. A complicated protein-ligand system in a water solvent system was created using Desmond/Maestro Schrödinger’s system builder. The system has a ten orthorhombic box form, and the volume was kept to a minimum. The water solvent was modelled using the simple point charge (SPC) water model. Nine Cl ions were used to neutralize the docked complex, and the system also received a 0.15 M addition of Na+ and Cl ions. At the first simulation, the entire system was at relaxed state. The system was then prepared for simulation in an isothermal-isobaric (NPT) ensemble at 310 k and 1.01325 bar pressure. The simulation time for molecular dynamics was 100 ns. OPLS-AA forcefield was used. RMSD values over the full-time span indicate less than 2-nm values (Fig. 4).

## Discussion

Dengue is a vector-borne viral disease that poses a significant public health challenge worldwide, with an estimated 390 million infections per year. Despite the significant burden of disease, there is currently no effective vaccine available to prevent dengue infection. The development of a safe and effective dengue vaccine is, therefore, an urgent priority [31–33]. Immunoinformatics is a rapidly evolving field that utilizes computational methods to predict and design immune-related molecules, such as epitopes, peptides, and vaccines. In this study, immunoinformatics was employed to explore the dengue (DENV-1) proteome to design a multi-epitope vaccine construct that can elicit a robust immune response against dengue infection. The study focused on identifying CD4+ epitopes in the DENV-1 proteome that can activate CD4+ T cells, which play a critical role in the adaptive immune response against viral infections. The CD4+ epitopes were identified using various bioinformatics tools, including the NetMHCIIpan server [34–37]. Here, we found four CD4+ epitopes NLKYSVIVTVHTGDQ, ANPIVTDKEKPVNIE, LDPVVYDAKFEKQL, and VGAIALDFKPGTSGS that were used to design vaccine construct. The identified CD4+ epitopes were then used to design a multi-epitope vaccine construct that can stimulate a broad and effective immune response against dengue infection. The vaccine construct included multiple CD4+ epitopes, as well as B-cell epitopes and T-cell epitopes. The results of this study demonstrate the potential of immunoinformatics approaches to identify and design multi-epitope vaccine constructs for dengue and other infectious diseases. The use of multiple epitopes in a single vaccine construct can enhance the breadth and potency of the immune response, leading to more effective protection against viral infections [38–40]. The use of immunoinformatics tools to design a multi-epitope vaccine construct based on CD4+ epitopes identified from the DENV-1 proteome is a promising strategy for the development of an effective dengue vaccine. The docking scores and RMSD value show the stable favorable interaction between TLR5 and vaccine construct. Further studies are needed to evaluate the efficacy and safety of this vaccine construct in preclinical and clinical trials.

## Conclusion

In conclusion, the use of immunoinformatics tools to explore the dengue (DENV-1) proteome and design a multi-epitope vaccine construct by using CD4+ epitopes is a promising approach to develop an effective dengue vaccine. The study demonstrated that the identified CD4+ epitopes from the DENV-1 proteome can activate CD4+ T cells, which play a critical role in the adaptive immune response against viral infections. The designed multi-epitope vaccine construct includes multiple CD4+ epitopes, B-cell epitopes, and T-cell epitopes, which can stimulate a broad and effective immune response against dengue infection. The approach of using multiple epitopes in a single vaccine construct can enhance the breadth and potency of the immune response, leading to more effective protection against viral infections. Moreover, the use of computational tools in immunoinformatics can accelerate the discovery and design of novel vaccine candidates. It enables the identification of potential epitopes that can stimulate the immune system without the need for extensive experimentation. In summary, the study provides a proof of concept for the use of immunoinformatics in the design of a multi-epitope vaccine construct for dengue and other infectious diseases. Further studies are needed to evaluate the efficacy and safety of the vaccine construct in preclinical and clinical trials. The developed approach can serve as a roadmap for the design of vaccines against other viral infections using immunoinformatics tools.

## Data Availability

All data is provided in manuscript.

## Abbreviations

RMSD: Root-mean-square deviation
CTL: Cytotoxic T lymphocyte
HTL: Helper T lymphocyte
MD: Molecular dynamics
DENV-1: Dengue virus 1 strain
